# Progress in PET Imaging of Neuroinflammation Targeting COX-2 Enzyme

**DOI:** 10.3390/molecules26113208

**Published:** 2021-05-27

**Authors:** Jaya Prabhakaran, Andrei Molotkov, Akiva Mintz, J. John Mann

**Affiliations:** 1 Department of Psychiatry, Columbia University Medical Center, New York, NY 10032, USA; jjm0@cumc.columbia.edu; 2 Department of Radiology, Columbia University Medical Center, New York, NY 10032, USA; am3355@cumc.columbia.edu (A.M.); am4754@cumc.columbia.edu (A.M.); 3 Area Molecular Imaging and Neuropathology, New York State Psychiatric Institute, New York, NY 10032, USA

**Keywords:** COX-2, neuroinflammation, BBB, PET imaging, radioligands

## Abstract

Neuroinflammation and cyclooxygenase-2 (COX-2) upregulation are associated with the pathogenesis of degenerative brain diseases such as Alzheimer’s disease (AD), Parkinson’s disease (PD), amyotrophic lateral sclerosis (ALS), epilepsy, and a response to traumatic brain injury (TBI) or stroke. COX-2 is also induced in acute pain, depression, schizophrenia, various cancers, arthritis and in acute allograft rejection. Positron emission tomography (PET) imaging allows for the direct measurement of in vivo COX-2 upregulation and thereby enables disease staging, therapy evaluation and aid quantifying target occupancy of novel nonsteroidal anti-inflammatory drugs or NSAIDs. Thus far, no clinically useful radioligand is established for monitoring COX-2 induction in brain diseases due to the delay in identifying qualified COX-2-selective inhibitors entering the brain. This review examines radiolabeled COX-2 inhibitors reported in the past decade and identifies the most promising radioligands for development as clinically useful PET radioligands. Among the radioligands reported so far, the three tracers that show potential for clinical translation are, [11CTMI], [11C]MC1 and [18F]MTP. These radioligands demonstrated BBB permeablity and in vivo binding to constitutive COX-2 in the brain or induced COX-2 during neuroinflammation.

## 1. Introduction

**Figure 1:**
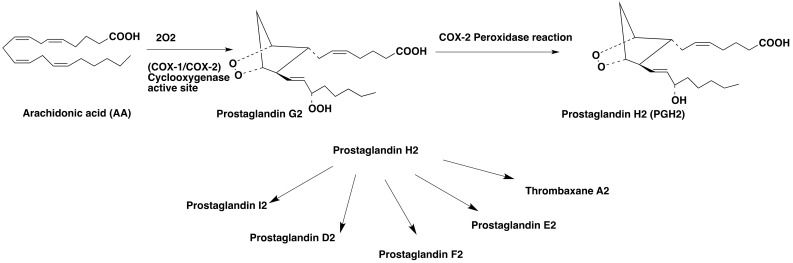
COX-2 mediated conversion of arachidonic acid into prostaglandin H2 (PGH2) and subsequent conversion of PGH2 into different eicosanoids.

Cyclooxygenase catalyzes the two-step biosynthetic conversion of arachidonic acid to prostaglandin H2 (PGH2) through its peroxidase activity (Figure 1) [1,2]. PGH2 is highly unstable, and it is subsequently converted to eicosanoids including other prostaglandins, prostacyclins, and thromboxanes. Prostaglandin E2 (PGE2) is the most abundant among these eicosanoids and it plays various physiological, homeostatic and pathological roles in the human body [3,4]. There are three different isoforms of COX expressed in humans, namely COX-1, COX-2, and COX-3 [5]. The constitutively expressed COX-1 is responsible for the maintenance of physiological functions such as gastrointestinal integrity, normal renal function, vascular homeostasis, and the biosynthesis of proaggregatory thromboxane A2 in blood platelets [5,6], whereas COX-2 is induced by growth factors and proinflammatory cytokines and it is responsible for prostaglandin production during inflammation. COX-3, however, is an alternative splicing variant of COX-1 found in the cerebral cortex and heart.

COX-1 and COX-2 show >60% homology between humans and rodents [2]. While the functional sites are conserved, conformational variations due to a few crucial substitutions in the 434 and 523 positions of the active site pockets contribute to their different affinities for specific inhibitors [7]. Both COX-1 and COX-2 are inhibited by NSAIDs, which have been used in the treatment of inflammation and pain for more than a century (e.g., aspirin and ibuprofen) [8,9]. However, the inhibition of constitutive COX-1 by non-selective NSAIDs causes gastrointestinal (GI) toxicity related to mucosa production and ulceration, and renal toxicity related to blood flow [10,11]. Consequently, several studies led to the development of COX-2-selective inhibitors (coxibs) with fewer gastrointestinal side effects. Coxibs showed improved GI safety compared with the non-selective NSAIDs. Hence, coxibs have been used for the treatment of arthritis, post-operative pain, headaches, as well as for inflammatory diseases of the brain and peripheral tissues [12,13]. Celecoxib and rofecoxib were the first FDA-approved coxibs used for the treatment of rheumatoid arthritis (RA), osteoarthritis (OA), and the relief of pain [14]. However, due to cardiovascular safety profiles, selective COX-2 inhibitors such as rofecoxib (Vioxx; COX-1/COX-2 = 272) and valdecoxib (Bextra; COX-1/COX-2 = 60) were withdrawn from the market in 2005 [15], whereas Celecoxib (Celebrex; COX-1/COX-2 = 30) is not reported to exhibit cardiovascular side effects compared with placebo and nonselective NSAIDs. Therefore, it remains an FDA-approved drug used for the treatment of RA, OA, and as a medication for pain relief [16]. Nonetheless, high affinity and selective coxibs can serve as imaging agents to study the underlying mechanism of inflammatory diseases associated with COX-2 induction and are also promising aids in the development of novel safe and effective coxibs.

### 1.1. Role of COX-2 in the Central Nervous System (CNS)

Although both COX-1 and COX-2 are expressed in the CNS and are associated with neuroinflammation, COX-2 is the predominant isoform found in the brain and spinal cord [17]. COX-2 is constitutively expressed in CNS and has effects on memory consolidation and functional hyperemia [18]. In the human brain, COX-2 is mainly expressed in the cerebral cortex, hippocampus, hypothalamus, midtemporal gyrus, substantia nigra, thalamus, caudate, cerebellum, and amygdala [19]. COX-2 induction plays a significant role in neurological disorders, which impact over 10% of the US population each year [20,21]. Therefore, the identification of effective diagnostic, preventive, and treatment strategies is of great importance for the management of neurological problems. Due to the significance of COX-2 in behavioral and cognitive functions and as a treatment target for brain diseases, measuring COX-2 levels in the brain is essential for medication selection and the monitoring of the treatment effect.

### 1.2. COX-2 Induction in the Pathognesis of CNS Diseases

Neuroinflammation and COX-2 upregulation are key features associated with the pathogenesis of many neurological diseases including brain ischemia, multiple sclerosis, epilepsy, AD and PD. COX-2 induction is also reported in psychiatric disorders such as major depressive disorders (MDD) and schizophrenia [22,23]. The elevation of COX-2 expression in AD is shown to be correlated with the severity of brain amyloid plaque pathology [24]. In addition, an increased level of COX-2 was found in all hippocampal layers of the human brain as the disease progressed to severe dementia [12,25,26]. Therefore, monitoring COX-2 induction in the brain, in vivo, may be an indicator of the progression of dementia as well as a target for therapy evaluation. Similarly, COX-2 upregulation is detected in dopaminergic neurons in PD, and the inhibition of COX-2 prevented the formation of dopamine–quinone associated with the pathogenesis of PD [27]. Hence, monitoring COX-2 induction in the brain may assist in the early diagnosis of PD and perform therapeutic evaluation targeting COX-2.

Although higher brain COX-2 expression was found in animal models of excitotoxicity, amyotrophic lateral sclerosis (ALS) and brain ischemia [28,29], the mechanism or biological alterations leading to epileptogenesis is unknown. The currently available antiepileptic drug therapy is sometimes ineffective or potentially causes severe side effects. It has also been reported that neuroinflammation and COX-2 induction contribute to the pathophysiology of seizure disorders [30]. Hence, COX-2 is considered to be a potential therapeutic target for epilepsy management. However, in vivo and noninvasive monitoring of COX-2 level is crucial to understand the extent of upregulation as well as the duration of maximum COX-2 induction to determine the effectiveness of COX-2-targeted therapy in seizure disorders.

### 1.3. Clinical Trials Using Coxibs in Neurological Disorders

Celecoxib was mainly used in the NSAID prevention trials of neurological diseases. However, due to low COX-2 affinity (IC_50_ = 40 nM) and limited brain uptake, celecoxib may not be the most ideal candidate for studies of brain diseases. Additionally, Dembo et. al demonstrated that celecoxib concentration in the cerebrospinal fluid of patients is lower than the dose required for therapeutic efficacy [31]. Therefore, the clinical trial results obtained in earlier studies cannot be generalized as the ineffectiveness of coxibs for preventing or treating neurological disorders. However, due to the lack of a successful tool for monitoring COX-2 in the living brain, the exact time-window for maximum COX-2 induction in neurological diseases was not measured. Besides, the target engagement of NSAIDs in neurological diseases should have been determined to predict treatment outcome. Therefore, PET imaging and the quantitative monitoring of COX-2 throughout the course of disease may be the most direct way of assessing the function of COX-2 in neurological diseases. Such a study would also help evaluate the appropriateness for inclusion in future NSAID prevention trials of neurological diseases.

## 2. In Vivo Measurement of Neuroinflammation

Neuroinflammation is a dynamic inflammatory response within the brain or spinal cord, which is mediated by cytokines, chemokines, reactive oxygen species and secondary messengers in response to trauma, infection and neuronal degeneration [32]. The degree of neuroinflammation depends on the context, duration and course of the primary stimulus [33]. Reliable in vivo measurement of neuroinflammation is essential to better characterize the inflammatory processes underlying various diseases and to aid the development of novel therapeutic agents. However, the development of new treatments for CNS disorders associated with neuroinflammation has been hampered due to the inadequate understanding of the in vivo biochemical mechanism involved. The lack of adequate tools to determine brain penetrance, target engagement, and molecular activity of novel drugs is also delaying drug development.

Human studies of neuroinflammation using PET imaging have been performed so far by measuring glucose metabolism using [18F]2-fluoro-2-deoxy-D-glucose (FDG) [34], PET imaging of trans-locator protein 18 kDa (TSPO) [35] and [11C]-labeled arachidonic acid [36]. However, glucose metabolism is modulated by many non-inflammatory factors, including neuronal activity, which limits the use of [18F]FDG for PET studies of neuroinflammation. Arachidonic acid, as well as other fatty acids, bind to phsopholipids and hence, they are not suitable to measure COX-2 induction associated with neuroinflammation. Another index of neuroinflammation is TSPO, but its presence in blood vessel walls and astrocytes should also be considered for accurate quantification. Hence, an increase in TSPO may not be purely a reflection of an inflammatory response [37]. Moreover, there is a need for genotyping before scanning to exclude those with lower affinity binding for most PET ligands. Additionally, it takes several days for the TSPO level to reach maximum density after a brain injury, which is then maintained for weeks even after the resolution of the inflammation. P2X7R, a ligand-gated ion channel belonging to the P2 receptor family, is widely expressed by different immune cells [38]. However, it is elevated in the brain only during early phases of the disease and the upregulation in neurological diseases such as ALS may not be sufficient to be detected by PET imaging. There are also a large number of active and inactive splice variants, or small nucleotide polymorphisms of P2X7R. Similarly, another known target for neuroinflammation is CB2, but its presence is found to be modest in neurological disorders [39]. This low expression may not be sufficient to quantify using PET imaging.

COX-2 is, therefore, considered one of the better targets for PET imaging neuroinflammation. COX-2 level is rapidly upregulated during inflammation and returns to baseline after few hours [40]. COX-2 inhibitors are a major class of anti-inflammatory drugs used in many medical conditions to reduce inflammation, thereby demonstrating the centrality of this enzyme in inflammation and measuring target occupancy for this class of therapeutics. Hence, the direct measurement of COX-2 levels in brain using PET imaging is important to measure changes in disease compared to controls.

### 2.1. Animal Models of Neuroinflammation

LPS is an important tool for studying neuroinflammation and has been used to produce a signal on COX-2 that is detectable by PET scanning [41]. The disadvantages of LPS models are that the inflammatory response depends on the serotype, route of administration, and number of injections of LPS. This model may also cause the disruption of many immune mediators and can be nonspecific for COX-2 overexpression. BBB is relatively resistant to LPS-induced disruption and up to 0.5 mg/kg of LPS i.v injection has shown to be optimum for inducing neuroinflammation without BBB compromise [42]. A high affinity and selective COX-2 radioligand would detect the upregulation of COX-2 in LPS models, and that can be confirmed by blocking studies using PET imaging. Alternative animal models such as a viral vector technology (adeno-associated virus (AAV)) may be used to induce the localized expression of COX-2 in rodents [43]. Alternatively, APP transgenic mice such as human (h)COX-2 transgenic mice with APPswe/PS1-A246E double-transgenic line mice with AD-like neuropathology can also be used [44].

### 2.2. PET Radioligands Developed for COX-2 Imaging in the Brain

PET is an in vivo method to detect biochemical changes with high sensitivity and can serve as a useful tool for studying neuroinflammation. High affinity and selective COX-2 inhibitors can be radiolabeled and examined for their in vivo binding to COX-2 in neuroinflammation. A successful radioligand thus identified can also be used for target occupancy and dose–response studies of novel NSAID medications using PET imaging. Multiple factors, such as LogD_7.4_ between 1 and 5, high target affinity, selectivity, no affinity to blood–brain barrier (BBB) active efflux transporters, kinetic profile, high dynamic range, suitable metabolic profile, the absence of brain penetrant metabolites, etc., determine the success of a PET radioligand designed for CNS imaging [45].

**Figure 2:**
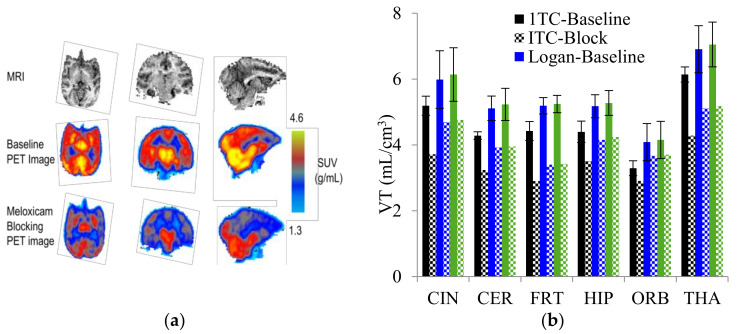
(**a**) MRI and summed (0–120 min) PET images of [^11^C]TMI in healthy baboon: (**b**) total volume of distribution (VTs) in baboon brain, baseline and blocking, estimated using a one-tissue compartment (1TC), Logan plot and likelihood estimation in graphical analysis (LEGA) methods. CIN: cingulate cortex; CER: cerebellum; FRT: frontal cortex; HIP: hippocampus; ORB: orbital cortex; THA: thalamus.

Radiolabeled arachidonic acid, the natural substrate of COX-2, was examined for measuring COX-2 induction in the brain; however, as mentioned above, this tracer was found to be not suitable due to its incorporation into phospholipids in the brain [46]. Additionally, several of the COX-2 tracers reported thus far showed poor uptake in target organs [47]. In general, low COX-2 affinity, high non-specific binding, the inability to passively transport through the BBB, and substantial defluorination in the case of [18F] tracers, etc. are other primary limitations of the earlier radiotracers. Table 1 describes the [11C] and [18F]-labeled coxibs developed in the past decade and results of their in vivo COX-2 imaging studies. Please note that all of the radioligands shown in Table 1 have arylsulfone moiety, providing better COX-2 selectivity [48]. Figure 2 shows PET images we obtained using an arylsulfone-based COX-2 inhibitor, [11C]TMI, with the highest COX-2 affiniy (<1 nM) and COX-1/COX-2 selectivity (>500,000). Although several tracers presented in Table 1 showed BBB permeability, their translation to PET studies in disease models is hampered by lack of sufficient binding in the brain or by complex radiosynthesis requiring heavy metals, leading to an inadequate amount of radioactive product and with desirable specific activity.

### 2.3. PET Studies of [11C]Celecoxib and [11C]Rofecoxib in Rodents:

Several [11C]-labeled ester and amide analogs derived from indomethacin were reported early in the decade (structures are not shown in Table 1). However, their COX-2 affinity was not sufficiently high enough to image the constitutive expression of COX-2 in the brain, or they were substrates for BBB active efflux pumps such as P-gp, or they had low brain-to-blood ratio. Subsequently, arylsulfone-based coxibs with high COX-2 affinity and COX-1/COX-2 selectivity were radiolabeled. For example, [11C]celecoxib (entry 1 of Table 1) [49,50,51,52] and [11C]rofecoxib (entry 5) [47,49], with demonstrated COX-2-inhibitory activity, were evaluated as radioligands for COX-2 imaging. In vitro autoradiography indicated the high specific binding of [11C]rofecoxib to the cerebellum and brainstem of a normal brain compared to [11C]celecoxib [50]. Although both of these radioligands indicated BBB permeability in accordance with their Log*p* values shown in Table 1, they failed to detect COX-2 expression in in vivo PET assays, likely due to the low level of constitutive COX-2 in a normal rodent brain. Similarly, in vitro autoradiography studies indicated the high binding of [11C]rofecoxib in the hippocampus of hypoperfusion-induced ischemia compared to the uninjured contralateral region. However, [11C]rofecoxib failed to detect COX-2 induction in living brains of the ischemia model using PET imaging. Hence, based on the above studies, [11C]celecoxib and [11C]rofecoxib may be useful only for PET studies outside the brain of rodents.

### 2.4. PET Studies of [11C]Celecoxib in Baboon

As discussed above, microPET imaging did not show the binding of [11C]celecoxib in a rodent brain. It is likely that the constitutive COX-2 expression may not be sufficient enough in the small rodent brain to be detected using PET imaging. However, we performed PET imaging in the brain as well as whole body biodistribution of [11C]celecoxib in baboons using PET, which indicated radioactivity uptake in the brain [51,52]. The liver showed the highest residence time and the gallbladder was the critical organ for [11C]celecoxib. Organ level internal dose assessment (OLINDA) estimates indicated that the maximum permissible single study dosage of [11C]celecoxib in humans is 1110 MBq for both males and females under the 21 CFR 361.1. At this point, it may remain to be demonstrated whether it can detect COX-2 upregulation in neuroinflammation in a human brain. However, the radiosynthesis of [11C]celecoxib is performed under Stille reaction conditions involving the use of heavy metals such as tin and palladium, which may be challenging for routine production in clinical settings. This tedious radiosynthesis procedure combined with the insufficient specific activity of the radioproduct discourages the use of [11C]celecoxib for clinical studies.

### 2.5. [18.F]-Labled Analogues of Celecoxib

There are two [18F]-labeled structural analogues of celecoxib reported, as shown in entries 2 and 3 of Table 1. [18F]celecoxib analogue-1 (entry 2) showed higher COX-2 affinity (IC_50_ = 1.7 nM) compared to its parent celecoxib (COX-2 IC_50_ = 40 nM) and was also found to be metabolically stable [53]. However, it may not be a viable PET ligand due to the complex electrochemical radiofluorination method required for the incorporation of [18F] label resulting in <2% yield. [18F]celecoxib analogue 2 (entry 3), on the other hand, is one among a series of novel fluorine-containing COX-2 inhibitors synthesized based on celecoxib–NBD, a fluorescent COX-2 imaging agent [54]. In vitro inhibitory data showed that compound **3** has a COX-2 IC_50_ of 0.36 μM with a COX-1/COX-2 ratio of approximately 300. In vivo PET studies are reported to use [18F]-labeled **3** in a human colorectal cancer model (HCA-7). Although radiotracer uptake into COX-2-expressing HCA-7 cells was high, no COX-2-specific binding was observed [54]. Additionally, PET studies using this radioligand for brain imaging are not reported to date.

### 2.6. PET Studies of [18F]Celecoxib in Baboons

We reported radiolabeling of one of the trifluoromethyl fluorine atoms in celecoxib itself via [18F]-labeleing using a bromine to [18F^−^] exchange reaction (entry 4). The radiolabeling proceeded with 10 +/− 2% yield (EOS), >99% chemical and radiochemical purities, and a specific activity of 120 +/− 40 mCi/μmol (EOB). Rodent PET studies showed bone labeling indicating in vivo de [18F]fluorination of [18F]celecoxib. Additionally, no binding was observed in the rat brain [55]. PET studies in baboon, however, indicated a lower rate of de [18F]fluorination compared with rodents and also minor radioactivity uptake was observed in the normal baboon brain [56]. However, as mentioned above, the exchange reaction is not capable of producing the labeled product in sufficient yield and specific activity. This, combined with defluorination, is a disadvantage of [18F]celecoxib for advancing to clinical PET studies of brain diseases.

### 2.7. PET Studies Using [11C]MOV

The synthesis and radiosynthesis of a methoxy analogue of valdecoxib, [(11)C]4-[5-(4-methylphenyl)-3-(trifluoromethyl)-1H-pyrazol-1-yl]benzenesulfonamide ([11C]MOV) was performed by our group (entry 6). MicroPET studies in male Sprague Dawley rats showed no uptake of radioactivity in the brain [56], whereas the binding of [11C]MOV, which was partially blocked in the heart and duodenum of normal rats by valdecoxib, was obtained using microPET imaging. [11C]MOV also indicated little uptake in a normal baboon brain. Perhaps, a neuroinflammation model expressing elevated level of COX-2 may show detectable uptake of [11C]MOV in the brain. However, based on the available data, [11C]MOV is not a promising radioligand for PET studies of the brain, but may be useful for PET imaging of peripheral inflammatory diseases.

### 2.8. PET Studies of [11C]TMI

Our continued efforts in radiolabeling arylsulfone-based coxibs resulted in the identification and radiolabeling of a coxib known as TMI with superior affinity and selectivity to COX-2 (IC_50_ = < 1 nM; entry 7, Table 1) [57,58,59]. We reported the synthesis, radiosynthesis and in vivo PET studies of [11C]TMI in nonhuman primates [58]. PET imaging in normal baboons was performed after the intravenous (i.v.) administration of [11C]TMI, which showed BBB penetration and a heterogeneous brain uptake and retention pattern (Figure 2). Moreover, the baseline binding was blocked using meloxicam, which indicates specific binding to COX-2 in the brain. [11C]TMI exhibits uptake even to constitutive COX-2 expression in the brain, which makes it a potential candidate for in vivo PET studies of COX-2 upregulation in neuroinflammation. Further experiments in animal disease models, as well as the evaluation of kinetic parameters, are needed to further the translation of [11C]TMI for clinical studies of neurological disorders associated with COX-2 induction.

### 2.9. PET Studies of [11C]MC1

Another arylsulfone-based radiolabeled coxib, [11C]MC1, (entry 8, Table 1) is reported to have subnanomolar affinity (IC50 = 3 nM) and excellent selectivity to COX-2 [60,61]. PET studies of [1]MC1 in healthy rhesus macaques at baseline and after the injection of LPS into right putamen were performed and the tracer showed binding to COX-2 in the brain. [11C]MC1 is also tested in the joints of patients with rheumatoid arthritis and the binding was compared with healthy individuals using PET imaging. The binding of [11C]MC1 improved on day 1 after LPS injection in monkey brain. The V_T_ value was reported to be increased by 32–42% after LPS injection on to the right putamen of monkeys [61]. This report indicates that [11C]MC1 may be a potential radioligand to image and quantify COX-2 upregulation in the brain during neuroinflammation and in joints in arthritis. Inflammation can result in increased blood flow in tissue and increase radiotracer delivery, meaning it is essential to demonstrate that an increase in binding represents a real increase in specific binding.

### 2.10. PET Studies of [18F]FMTP

As described in the above sections, two highly potent coxibs, [11C]TMI and [11C]MC1, have demonstrated COX-2 binding in brain using in vivo PET imaging. However, the availability of an [18F]-labeled COX-2 tracer would have additional benefits in clinical studies due to its 110 min half-life. For example, [18F]-tracers allow one to scan for a longer duration to enable equilibrium binding and accomplish accurate kinetic modeling as well as the quantification of COX-2 binding. Additionally, [18F]-tracers can be transported off-site from a central production site to allow cost-effective multi-center clinical studies.

**Figure 3:**
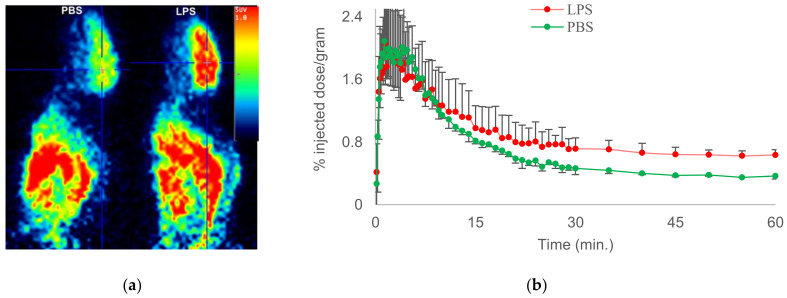
Representative microPET images of [18F]MTP in PBS- and LPS-administered mice (**a**); TACs of [18F]MTP in PBS- (green) and LPS (red)-treated mice (**b**).

We therefore reported [18F]-6-fluoro-2-(4-(methylsulfonyl)phenyl)-N-(thiophen-2-ylmethyl)pyrimidin-4-amine ([18F]FMTP), which is a fluoro-analogue of MCI with subnanomolar affinity to COX-2 (entry 9) [62]. The radiochemical synthesis and evaluation of [18F]MTP was optimized using a chlorine-to-fluorine displacement method, by reacting [18F]fluoride with the cholo-MTP precursor molecule [62]. PET imaging in wild-type mice indicated BBB penetration and the fast washout of [18F]MTP in the brain, likely due to the low constitutive COX-2 expression in normal mouse brain. LPS administered mice after tail vein administration of [18F]MTP showed a higher binding of radioactivity in the brain, indicating its potential to detect COX-2 induction in neuroinflammation (Figure 3). Hence, COX-2-specific binding measured in vitro, BBB permeability, and increased brain uptake in mice neuroinflammation qualifies [18F]MTP for further evaluation. An additional quality of [18F]MTP is that microPET images do not show the bone uptake of radioactivity, indicating the absence or slow rate of defluorination. This may be attributed to the [18F]-label on the aromatic ring, which does not undergo fast defluorination under normal physiological conditions. PET studies in animal models of neurological disorders are underway to further characterize [18F]MTP as a potential candidate for COX-2 imaging in clinical studies of neurological diseases.

## 3. Conclusions

COX-2 is an important target for the PET imaging of neuroinflammation in neurodegenerative diseases. As described in Section 2, there are drawbacks associated with targets such as TSPO, P2X7R and CB2 for neuroinflammation imaging. COX-2 is, therefore, considered one of the better targets for PET imaging neuroinflammation. It is rapidly upregulated by inflammation and returns back to baseline levels within few hours. Hence, studying the time-dependent expression profile of COX-2 may be necessary to assess the highest level of COX-2 induction during a disease process. Many of the currently available radioligands have limitations for PET imaging of the brain COX-2 due to insufficient affinity and selectivity, lack of BBB permeability, or complex radiosynthesis procedure leading to inconsistent yield and specific activity of the radioproduct. This review summarises the various radiolabeled coxibs that have been synthesized and evaluated during this time and examines the most suitable structural core for in vivo imaging COX-2 induction in the living brain. All of the radioligands presented in Table 1 have an arylsulfone component, which is a common functional moiety for preferential COX-2 binding. Among these, [11C]MC1, [11C]TMI, and [18F]MTP have sub-nanomolar COX-2 affinity, superior COX-1/COX-2 selectivity, and Log*p* value < 4. These radioligands showed BBB permeability and in vivo binding to COX-2 in brain and have also been tested in LPS models of neuroinflammation by PET imaging. LPS models have shown high reproducibility in the inflammatory response elicited and are suitable models for the initial in vivo evaluation of COX-2 radioligands. Thus, having demonstrated in vivo binding to COX-2 in the brain, the mentioned ligands qualify for further investigation in animal models of diseases and potential translation for clinical imaging. A successful PET ligand would be applicable for assisting early diagnosis, monitoring therapeutic response, and aid the development of new NSAID medications by dose response studies.

## Figures and Tables

**Table 1:** [11C] and [18F]-labeled radioligands developed in the past decade for PET imaging COX-2.

Entry	PET Ligand	COX-2 IC_50_	COX-1/COX-2	Log*P*	In Vivo Binding in Brain
1	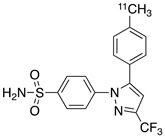 [11C]celecoxib	40 nM	30	3.6	PET imaging did not show binding in rodent brain. However, low binding was found in healthy baboon brain, indicating BBB permeability. PET studies in inflammation model are not reported [49,50,51,52].
2	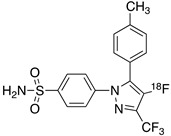 [18F]celecoxib analogue 1	1.7 nM	224	3.3	No in vivo binding is reported in rodent brain. PET imaging in other species or neuroinflammation model are not reported [53].
3	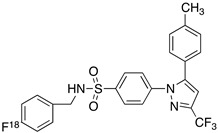 [18F]celecoxib analogue 2	360 nM	278	5.8	In vivo binding was not reported in rodent brain. PET imaging in other species or in neuroinflammation model are not reported [54].
4	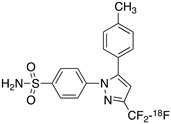 [18F]celecoxib (**4**)	40 nM	30	3.7	Significant bone uptake was detected in rodent PET imaging. Minor binding was observed in normal baboon brain, which indicates BBB penetration. We detected less skeletal uptake in baboon compared to rodents [55].
5	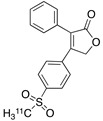 [11C]rofecoxib (**5**)	19 nM	272	1.8	In vivo binding of the tracer is not reported in rodent brain. PET imaging in other species or in neuroinflammation models are also not reported [47,49].
6	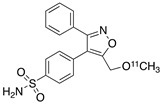 [11C]MOV (**6**)	35 nM	342	1.6	There was no detectable binding in rat brain. Binding in healthy baboon brain was also not significant. Therefore, no neuroinflammation model was tested [56].
7	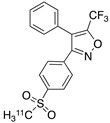 [11C]TMI (**7**)	1 nM	500,000	2.7	Moderate binding was detected in brain by PET imaging in normal baboon. The binding was blocked using meloxicam [57,58,59].
8	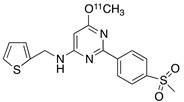 [11C]MC1 (**8**)	3 nM	100,000	2.6	The V_T_ value was increased by 32–42% after LPS injection on to the right putamen of monkeys compared to healthy monkeys [60,61].
9	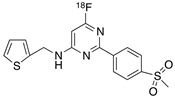 [18F]MTP (**9**)	2.3 nM	52,000	2.6	Although the baseline binding in normal rodent brain was low, a higher binding was observed in LPS administered mice brain. No bone uptake was detected [62].

## Data Availability

The data presented in this review are available in the corresponding publications listed in the reference section.

## Significance Statement

The COX-2 isoenzyme plays a crucial role in the inflammatory pathogenesis of neurological disorders, stroke, cancers, arthritis, organ rejection and pain. PET imaging using radiolabeled COX-2 inhibitors would permit the noninvasive quantification of COX-2 in brain and facilitate disease staging, therapy evaluation, and the development of new anti-inflammatory medications targeting brain COX-2. The radioligands reported over the past decade for COX-2 imaging, and the most suitable tracers among them for further investigation in PET studies of neuroinflammatory diseases, are presented in this review.
